# Impact of Dry Mouth and Factors Associated with Sarcopenia on Oral Health-Related Quality of Life in Peritoneal Dialysis Patients

**DOI:** 10.1055/s-0045-1802567

**Published:** 2025-03-12

**Authors:** Natcha Boonyapratheeprat, Kununya Pimolbutr, Dulyapong Rungraungrayabkul, Sasiwimon Meenetkum, Sarinya Boongird, Piyatida Chuengsaman, Nis Okuma, Supanee Thanakun, Chagriya Kitiyakara, Sujiwan Seubbuk Sangkhamanee

**Affiliations:** 1 Department of Oral Medicine and Periodontology, Faculty of Dentistry, Mahidol University, Bangkok, Thailand; 2 Department of Epidemiology, Faculty of Public Health, Mahidol University, Bangkok, Thailand; 3 Department of Medicine, Faculty of Medicine Ramathibodi Hospital, Mahidol University, Bangkok, Thailand; 4 Banphaeo-Charoenkrung Peritoneal Dialysis Center, Banphaeo Dialysis Group, Banphaeo Hospital, Bangkok, Thailand; 5 Division of Oral Diagnostic Science, College of Dental Medicine, Rangsit University, Pathum Thani, Thailand

**Keywords:** end-stage renal disease, oral health-related quality of life, sarcopenia, xerostomia

## Abstract

**Objectives:**

This cross-sectional study aimed to investigate the oral health-related quality of life (OHRQoL), its associated factors, and the prevalence of possible sarcopenia in Thai well-maintained patients with end-stage renal disease (ESRD) undergoing peritoneal dialysis (PD).

**Materials and Methods:**

Data were collected from 63 participants undergoing PD at Banphaeo-Charoenkrung Hemodialysis Center. Dry mouth was evaluated through unstimulated salivary flow rate measurement and self-reported xerostomia questionnaires. OHRQoL was assessed using the Thai version of Oral Health Impact Profile (OHIP-14).

**Statistical Analysis:**

Statistical analyses were conducted using IBM SPSS Statistics version 21.0. Descriptive statistics summarized participant characteristics, and normality was tested with the Kolmogorov–Smirnov test. Continuous variables were expressed as medians and interquartile ranges, while categorical variables were presented as frequencies and percentages. The Mann–Whitney *U* test and Fisher's exact test were used to assess differences between OHRQoL groups. Partial Spearman's rank correlation examined variable relationships, and logistic regression identified factors linked to a higher negative impact on OHRQoL, adjusting for age, sex, body mass index, chair stand test, and salivary flow rate. A *p* -value of < 0.05 was considered significant.

**Results:**

The median age was 59 years (range 27–79), with a possible sarcopenia prevalence of 52.4%. OHIP-14 scores ranged from 0 to 32, with medians of 4 and 13 in a lower ( *n* = 31) and higher ( *n* = 32) negative impact on OHRQoL, respectively. Those with a higher negative impact on OHRQoL exhibited a significantly higher proportion of self-reported xerostomia ( *p* = 0.01), lower salivary flow rate ( *p* = 0.01), and longer 5-time chair stand test ( *p* = 0.04) compared to individuals with the lower negative impact on OHRQoL. Correlation between the time of the chair stand test and the handgrip strength adjusting for age ( *r* = –0.439, *p* < 0.001) and sex ( *r* = –0.351, *p* = 0.006) was revealed. Multivariate logistic regression showed a significant association between salivary flow rate and a higher negative impact on OHRQoL (odds ratio 0.018; 95% confidence interval: 0.001, 0.545; *p* = 0.02).

**Conclusion:**

This finding suggests that reduced salivary flow affected OHRQoL in well-maintained ESRD patients with PD, highlighting the importance of managing dry mouth to alleviate their OHRQoL.

## Introduction

Chronic kidney disease (CKD), characterized by impaired kidney function and/or a reduced glomerular filtration rate, is a significant public health concern worldwide. 1 In Thailand, CKD affects between 4.7 and 11.2% of the population. 1 Since the leading causes of CKD are common metabolic syndromes, including hypertension and diabetes, the prevalence of CKD is expected to continue to rise. 1 2 Peritoneal dialysis (PD), alongside hemodialysis (HD) and renal transplantation, serves as a primary treatment modality for end-stage renal disease (ESRD), the final stage of CKD. 3 Despite advancements in medical care, ESRD patients still suffer from increased mortality and reduced functional ability.

CKD patients face various physiological and psychological challenges, which adversely affect their quality of life. 2 These challenges are exacerbated as CKD progresses to ESRD. 4 Additionally, CKD is associated with compromised oral health, 4 5 with ESRD patients frequently experiencing dry mouth and halitosis. 6 Evidence indicates that CKD patients often suffer from decreased salivary flow rates, increased saliva viscosity, altered pH, and reduced buffer capacity. 7 8 These alterations contribute to xerostomia and associated oral health issues, including difficulties in chewing, swallowing, and speaking. 9 Oral health-related quality of life (OHRQoL) encompasses an individual's well-being concerning oral function, emotional impact, and overall satisfaction. 10 Studies have shown that CKD patients have lower OHRQoL than healthy individuals. 4 6 11 12 Rodakowska et al 12 reported that the most affected OHRQoL index categories in chronic HD patients ranged from psychological impact, pain and discomfort, behavioral impact, and functional limitation. The OHRQoL of patients undergoing renal replacement therapy is also thought to negatively impact oral health and disease-related factors. 4

Sarcopenia, characterized by age-related loss of lean skeletal muscle mass, is prevalent among CKD patients, particularly those with ESRD. 13 The Asian Working Group for Sarcopenia (AWGS) introduced screening for possible sarcopenia to enable earlier lifestyle interventions for community-based health promotion. 14 Possible sarcopenia is defined by either low physical performance or low muscle strength, 14 15 which is associated with adverse clinical outcomes such as mortality, falls, and dependency in the elderly. 16 17 Takahashi et al. found that elderly patients with sarcopenia exhibited lower OHRQoL than healthy patients. 17 Reduced OHRQoL is also a predictor of long-term outcomes in ESRD patients. 18 However, data on OHRQoL in ESRD patients undergoing PD is limited.

Therefore, this study aimed to investigate OHRQoL in Thai well-maintained ESRD patients with PD and its associated factors. Moreover, the research sought to evaluate the prevalence of possible sarcopenia in terms of physical performance and muscle strength. Insights gained from this research may enhance optimizing care in this high-risk population.

## Materials and Methods

### Study Population

Sixty-three ambulatory participants were recruited from well-maintained ESRD patients undergoing PD at Banphaeo-Charoenkrung Hemodialysis Center based on convenience sampling. The inclusion criteria were patients aged 25 to 80 years diagnosed with ESRD according to the Kidney Disease: Improving Global Outcomes (KDIGO) Clinical Practice Guideline 2012 and currently receiving PD. 19 Exclusion criteria included a history of kidney transplantation, a positive coronavirus disease 2019 (COVID-19) test, and conditions contraindicating physical examination (e.g., ventilator use, intravenous nutrition, nasogastric tube, bedbound status, amputated limbs, or metallic devices like cardiac pacemaker). Informed consent was obtained, and the study adhered to the Declaration of Helsinki, with approval from the Human Research Ethics Committee, Faculty of Medicine Ramathibodi Hospital, Mahidol University Bangkok, Thailand (COA.MURA2022/719).

### Data Collection

Participants' characteristics, including age, sex, employment status, education, CKD causative diseases (e.g., diabetes, hypertension), and medications, were collected. Weight and height were measured to calculate body mass index (BMI).

### Assessment of Oral Health-Related Quality of Life

OHRQoL was assessed using the Oral Health Impacts Profile-14 (OHIP-14), employing the validated Thai version. 10 The OHIP-14 questionnaire comprises seven subscales: functional limitation, physical pain, psychological discomfort, physical disability, psychological disability, social disability, and handicap, each with two questions. Responses were rated on a Likert scale reflecting frequency over the past 4 weeks: Always = 4, Often = 3, Sometimes = 2, Seldom = 1, and Never = 0. The total OHIP-14 score, ranging from 0 to 56, was calculated by summing the responses. Higher scores indicate poorer OHRQoL. In this study, participants were categorized into two groups based on the median OHIP-14 score: lower (total OHIP-14 scores < 8) and higher (total OHIP-14 scores of ≥ 8) negative impact on OHRQoL.

### Assessment of Dry Mouth

The participants were asked to provide a saliva sample over 15 minutes using the spitting method to assess the unstimulated salivary flow rate. 20 Before saliva collection, participants were instructed to refrain from eating and drinking for at least 2 hours. The cutoff value for the unstimulated salivary flow rate for diagnosing as objective dry mouth or hyposalivation is ≤ 0.1 mL/min. 21

Xerostomia was evaluated using the self-reported questionnaire of four questions from Fox. 22 The diagnosis of xerostomia was determined if at least one question yielded a positive response.

### Assessment of Sarcopenia

Participants were screened for possible sarcopenia in a community preventive service setting according to the AWGS 2019 criteria, using a 5-time chair stand test and handgrip strength measurement to assess physical performance and muscle strength, respectively. 14 The chair stand test involved a straight-backed chair with a 16-inch seat height. Participants, with arms folded across their chest, were instructed to stand up and sit down as quickly as possible for five repetitions, and the time taken was recorded in seconds. A completion time of ≥ 12 seconds indicated low physical performance for both sexes. Handgrip strength was measured with a digital dynamometer (T.K.K.5401 GRIP-D, Takei Scientific Instruments Co., Ltd., Tokyo, Japan), with participants exerting maximum force twice with each hand and the average value was recorded. Low muscle strength was defined as < 28 kg for men and < 18 kg for women. 14 Participants exhibiting either low physical performance or low muscle strength were classified as having possible sarcopenia.

### Statistical Analysis

Statistical analyses were performed using a statistical software package (IBM SPSS Statistics version 21.0; SPSS Inc., Armonk, New York, United States). Descriptive analyses were employed to describe patient characteristics. Kolmogorov–Smirnov test was utilized to evaluate normal distribution. Age, BMI, salivary flow rate, total OHIP-14 score, chair stand test, and handgrip strength were reported as the median and interquartile range (IQR). Sex, underlying diseases, current medications, and self-reported xerostomia were reported as frequency and percentage.

Differences or associations in the parameters mentioned above, according to patients with a lower and higher negative impact on OHRQoL, were assessed using the Mann–Whitney *U* test or Fisher's exact test as appropriate. Moreover, partial Spearman's rank correlation was used to examine the correlation between the variables of interest. Logistic regression models were performed to investigate the factors related to the higher negative impact on OHRQoL. The multivariate model was adjusted for age, sex, BMI, chair stand test, and salivary flow rate. A *p* -value of < 0.05 was considered statistically significant.

## Results

### Patient Characteristics

Sixty-three ESRD patients undergoing PD, aged 27 to 79, were recruited. The median age of participants was 59 (IQR = 52, 68). Males (61.9%) were more prevalent than females (38.1%). The median BMI of all participants was 23.4 (IQR = 20.8, 27.0). The proportion of employed participants (50.8%) was similar to those who were unemployed or retired (49.2%). Most participants graduated ( *n* = 47, 74.6%) from secondary school.

### Patient Characteristics and OHRQoL Status

The baseline characteristics of all participants with different OHRQoL status are shown in Table 1 . In these ESRD patients, the OHIP-14 score ranged from 0 to 32. The median total OHIP-14 score in the higher negative impact on the OHRQoL group was statistically significantly higher than in the lower negative impact on the OHRQoL group ( *p* < 0.001). Thirty-one (49.2%) and 32 (50.8%) participants had lower negative OHRQoL and higher negative OHRQoL, respectively. Most participants (95.2%) received at least one medication that could influence salivary flow. Nevertheless, the two groups showed no significant differences in age and BMI. Sex, occupation, education, underlying diseases, and related medication were not associated with the status of OHRQoL.

**Table 1:** Characteristics of participants according to lower and higher levels of negative impact on OHRQoL

Variables	Negative impact on OHRQoL		*p* -Value
	Lower ( *n* = 31)	Higher ( *n* = 32)	
Age [Median (IQR)]	57 (53.0, 67.0)	61.5 (50.5, 68.0)	0.67 a
Sex [ *n* (%)] • Male • Female	17 (54.8) 14 (45.2)	22 (68.8) 10 (31.2)	0.31 b
BMI (kg/m ^2^ ) [Median (IQR)]	23.2 (21.8, 27.3)	24 (20.8, 26.6)	0.97 a
Occupation [ *n* (%)] • Employed • Unemployed or retired	15 (48.4) 16 (51.6)	17 (53.1) 15 (46.9)	0.80 b
Education [ *n* (%)] • ≤ secondary school • > secondary school	25 (80.6) 6 (19.4)	22 (68.8) 10 (31.2)	0.39 b
Causative diseases of CKD			
Diabetes [ *n* (%)] • No • Yes	15 (48.4) 16 (51.6)	15 (46.9) 17 (56.1)	1.00 b
Hypertension [ *n* (%)] • No • Yes	4 (12.9) 27 (87.1)	4 (12.5) 28 (87.5)	1.00 b
Taking at least one medication associated with xerostomia [ *n* (%)] • No • Yes	2 (6.5) 29 (93.5)	1 (3.1) 31 (96.9)	0.61 b
Total OHIP-14 [Median (IQR)]	4 (2, 5)	13 (10, 19.5)	** < 0.001 a**

Abbreviations: BMI, body mass index; CKD, chronic kidney disease; IQR, interquartile range; OHIP-14, Oral Health Impacts Profile-14; OHRQoL, oral health-related quality of life.

Note: Bold value indicates statistically significant ( *p* < 0.05).

a Mann–Whitney *U* test.

b Fisher's exact test.

### Dry Mouth, Sarcopenia, and OHRQoL Status

In the present study, the overall prevalence of xerostomia was 52.4% ( *n* = 33). Patients in the higher negative impact OHRQoL group exhibited a significantly higher proportion of xerostomia compared with those in the lower negative impact OHRQoL group ( *p* = 0.01) ( Table 2 ). When assessing unstimulated salivary flow, the prevalence of hyposalivation was 12.7% ( *n* = 8). The average salivary flow rate in the patients with xerostomia was 0.27 mL/min, whereas that of individuals without xerostomia was 0.38 mL/min ( *p* = 0.01). The average unstimulated salivary flow rate in those with a higher negative impact on OHRQoL was significantly lower than in those with a lower negative impact on OHRQoL ( *p* = 0.01).

**Table 2:** Participants' dry mouth and possible sarcopenia status according to lower and higher levels of negative impact on OHRQoL

Parameters	Negative impact on OHRQoL		*p* -Value
	Lower ( *n* = 31)	Higher ( *n* = 32)	
Self-reported xerostomia [ *n* (%)] • No • Yes	20 (64.5) 11 (35.5)	10 (31.3) 22 (68.8)	** 0.01 a**
Salivary flow rate [Median (IQR)]	0.42 (0.27, 0.54)	0.27 (0.17, 0.34)	** 0.01 b**
5-time chair stand test [Median (IQR)]	11 (10, 14.25)	14 (11, 21)	** 0.04 b**
Handgrip strength [Median (IQR)] • Male • Female	24.7 (16.2, 30.0) 28.3 (23.0, 37.0) 16.4 (14.7, 26.5)	22.4 (16.5, 28.8) 26.2 (19.4, 31.2) 17.2 (13.1, 22.1)	0.30 b 0.34 b 0.30 b

Abbreviations: IQR, interquartile range; OHRQoL, oral health-related quality of life.

Note: Bold values indicate statistically significant ( *p* < 0.05).

a Fisher's exact test.

b Mann–Whitney *U* test.

The prevalence of possible sarcopenia in this study was 52.4% ( *n* = 33). The 5-time chair stand test demonstrated that the group with a higher negative impact on OHRQoL had low physical performance, taking significantly longer to complete the test than the group with a lower negative impact on OHRQoL ( *p* = 0.04). However, the handgrip strength did not differ significantly between the two groups. Noticeably, except for males with a lower negative impact on the OHRQoL, average handgrip strengths were below the normal range for both sexes ( Table 2 ). In addition, there was a significant correlation between time of chair stand test and the handgrip strength adjusting for age ( *r* = –0.439, *p* < 0.001) and sex ( *r* = –0.351, *p* = 0.01), respectively.

### Factors Associated with the Higher Negative Impact on OHRQoL

Table 3 demonstrates factors associated with the higher negative impact on OHRQoL in the univariate and multivariate logistic regression analysis. The univariate analysis showed that the salivary flow rate was significantly associated with an increased risk of having a higher negative impact on OHRQoL (odds ratio [OR] = 0.011, 95% confidence interval [CI] 0.001, 0.335, *p* = 0.01). After adjusting for all potential confounding factors in the multivariate analysis, salivary flow rate remained a significant factor associated with a higher negative impact on OHRQoL (OR = 0.018, 95% CI 0.001, 0.545, *p* = 0.02). In other words, for every 0.1 mL/min increase in the salivary flow rate, there was a 33% decreased risk of a higher negative impact on OHRQoL. However, age, sex, BMI, and chair stand test were not associated with a higher negative impact on OHRQoL.

**Table 3:** Logistic regression analyses on associations of associated factors and a higher level of negative impact on OHRQoL

	Higher level of negative impact on OHRQoL			
	Unadjusted OR (95% CI)	*p* -Value	Adjusted OR a (95% CI)	*p* -Value
Age	1.02 (0.98, 1.06)	0.40	0.99 (0.94, 1.04)	0.55
Sex Male Female	1 1.72 (0.60, 4.89)	0.31	1 2.73 (0.82, 9.13)	0.10
BMI	0.98 (0.87, 1.11)	0.78	0.96 (0.83, 1.10)	0.52
Chair stand test	1.11 (1.01, 1.22)	0.14	1.10 (0.97, 1.25)	0.14
Salivary flow rate (mL/min)	0.011 (0.001, 0.335)	**0.01**	0.018 (0.001, 0.545)	**0.02**

Abbreviations: BMI, body mass index; CI, confidence interval; OHRQoL, oral health-related quality of life; OR, odds ratio.

Note: Bold values indicate statistically significant ( *p* < 0.05).

a Adjusted for age, sex, BMI, chair stand test, and salivary flow rate.

## Discussion

The present study aimed to investigate OHRQoL and its associated factors among Thai ESRD patients with PD. The findings reveal that a reduced salivary flow rate was associated with a higher level of negative impact on OHRQoL (OHIP-14 ≥ 8). Moreover, the study provides insights into the prevalence of possible sarcopenia within this patient group.

The assessment of OHRQoL employed the Thai OHIP-14, chosen for its established reliability and widespread use in evaluating OHRQoL among Thai adolescents. 10 Notably, patients in our study demonstrated superior OHRQoL compared to the previous report in Thai PD patients. 18 This observation may be attributed to the high standard of care at the Banphaeo-Charoenkrung Hemodialysis Center, which features a well-structured system for regular follow-up and high treatment compliance. Prior research indicated that PD patients tend to demonstrate relatively better OHRQoL than their HD counterparts. 23 24 Nevertheless, even well-maintained PD patients still experience a more pronounced negative impact on OHRQoL compared to community-dwelling elders. 25

Regarding xerostomia, a subjective symptom often emphasized in patients with dry mouth, the study found a significant prevalence of 52.4% among ESRD patients, consistent with previous research (40.0–74.2%). 26 27 28 This prevalence surpasses that of the general population (0.9–64.8%). 29 The proportion of self-reported xerostomia was significantly higher in ESRD patients, who had a higher negative impact on OHRQoL. Hyposalivation, on the other hand, refers to the objective finding of reduced salivary flow rate. Both xerostomia and hyposalivation frequently impairs eating, swallowing, and speaking abilities 9 and significantly affects OHRQoL. 30 31 Furthermore, they are associated with an increased risk of dental caries, periodontal disease, and oral candidiasis, all of which affect OHRQoL. 32 33 It is important to note, however, that xerostomia does not always correlate with decreased salivary flow, as other factors, such as changes in salivary composition, 34 may also contribute. Nonetheless, our findings demonstrated that the salivary flow rate in ESRD patients correlated with self-reported xerostomia and was associated with a higher negative impact on OHRQoL. Interestingly, only the effect of low salivary flow rate on OHRQoL persisted after adjusting for age. Moreover, the impact of reduced salivary flow on OHRQoL aligns with findings in elderly individuals without ESRD, 35 36 37 although the participants in our study spanned a wide age range (from 27 to 79 years), suggesting that aging may not be a major contributing factor in our study. Additionally, while the majority of participants in our study were on medications known to affect salivary flow, no significant differences were observed between those with lower or higher negative impacts on OHRQoL. This suggests that the deterioration in OHRQoL among ESRD patients is more likely attributed to hyposalivation caused by the ESRD itself, rather than by medications. These findings underscore the importance of regular evaluation and management of dry mouth in ESRD patients undergoing PD to mitigate its impact on oral health. Addressing salivary dysfunction is crucial to prevent further deterioration in oral health and overall quality of life in this vulnerable population.

Sarcopenia, a consequence of poor nutrition, physical inactivity, and increased protein degradation, 38 affects elderly individuals with dysphagia and poor chewing ability, potentially impairing oral health function. 16 The interplay between oral health, malnutrition, and features of sarcopenia, driven by inflammation and oxidative stress, may impact OHRQoL in CKD patients. 13 In this study, one-half of the ESRD patients on PD exhibited possible sarcopenia. Previous research reports a wide prevalence of sarcopenia in PD patients, from 2.2 to 75.6%, influenced by varying assessment criteria and methods. 13 Contributing factors include ESRD status, comorbidities, and low physical activity, leading to muscle loss regardless of age. A significant difference in the 5-time chair stand test was noted between OHRQoL groups, with those experiencing a higher negative impact on the OHRQoL showing low physical performance or possible sarcopenia. However, the logistic regression did not reveal a significant association between this test and OHRQoL. In addition, the 5-time chair stand test significantly correlated with handgrip strength independent of age and sex, consistent with findings from a Korean cohort study. 39 suggesting its potential utility in assessing possible sarcopenia. Further research with larger populations is necessary to better understand the relationship between these measures and OHRQoL.

The study utilized the validated Thai OHIP-14 10 to assess OHRQoL, with data collected through native-language interviews. The questionnaires effectively covered various aspects of life and demonstrated validity and reliability for evaluating the impact of oral health issues. 40 Both subjective and objective symptoms of dry mouth were assessed. Nevertheless, this study is limited by its sample size, which was small, constrained by the COVID-19 pandemic, and its cross-sectional design, which precludes establishing causal relationships between oral health-associated factors, sarcopenia, and OHIP-14. The lack of oral examination, due to the setting, is another limitation. Future research should incorporate oral examination and multicenter longitudinal studies with larger sample sizes to validate these findings and further explore OHRQoL in Thai ESRD patients undergoing PD.

## Conclusion

In conclusion, this study underscores the significant impact of low salivary flow rate on the OHRQoL of ESRD patients on PD and highlights prevalent issue of possible sarcopenia for its tendency with negative OHRQoL. These preliminary results emphasize the need to address oral health comprehensively in the care of ESRD patients on PD to improve their overall quality of life. Future research should focus on investigating these associations more thoroughly to develop effective interventions.
